# Autoantibody Response to Murine Double Minute 2 Protein in Immunodiagnosis of Hepatocellular Carcinoma

**DOI:** 10.1155/2014/906532

**Published:** 2014-05-14

**Authors:** Mei Liu, Su-jun Zheng, Yu Chen, Ning Li, Peng-fei Ren, Li-ping Dai, Zhong-ping Duan, Jian-Ying Zhang

**Affiliations:** ^1^Beijing You'an Hospital, Capital Medical University, Beijing 10069, China; ^2^Department of Biological Sciences, The University of Texas at El Paso, El Paso, TX 79968, USA

## Abstract

Hepatocellular carcinoma (HCC) is the fifth most common malignancy worldwide. Although new therapeutic strategies have been continuously developed and applied to clinical treatment for HCC, the prognosis is still very poor. Thus, early detection of HCC may enhance effective and curative management. In this study, autoantibody responses to MDM2 protein in HCC patient's serum were evaluated by enzyme-linked immunosorbent assay (ELISA) and part sera were evaluated by Western blotting and indirect immunofluorescence assay. Immunohistochemistry (IHC) over tissue array slides was also performed to analyze protein expression of MDM2 in HCC and control tissues. The prevalence of autoantibodies against MDM2 was significantly higher than that in liver cirrhosis (LC), chronic hepatitis (CH), and normal human sera (NHS). The average titer of autoantibodies against MDM2 in HCC serum was higher compared to that in LC, CH, and NHS. A high titer of autoantibodies against MDM2 in ELISA could be observed in the serum in 6 to 9 months before the clinical diagnosis of HCC in the serum of several HCC patients with serial bleeding samples. Our preliminary data indicate that MDM2 and anti-MDM2 system may be a potential biomarker for early stage HCC screening and immunodiagnosis.

## 1. Introduction

Hepatocellular carcinoma (HCC) is the fifth most common malignancy worldwide. It represents the fifth most prevalent cancer worldwide and accounts for 500 000 deaths each year [1, 2]. HCC is associated with a poor prognosis due to a lack of effective treatment options. Although new therapeutic strategies have been continuously developed and applied to clinical treatment of HCC, the prognosis is still very poor. It has a survival rate of less than 5% and an average survival of less than one year after diagnosis [3]. There is still no effective therapy for most patients with advanced or metastatic HCC [4]. Early detection of HCC enhances effective and curative management. The sensitivity and specificity of serum alpha-fetoprotein (AFP) in HCC diagnosis are not optimal. In recent years tumor-associated antigens (TAAs) were studied by researchers in order to find better early stage biomarker of HCC.

The MDM2 oncogene, biochemically as E3 ubiquitin protein ligase, was originally identified by virtue of its amplification in a spontaneously transformed derivative of mouse BALB/c cells and the MDM2 protein subsequently was shown to bind to p53 in rat cells transfected with p53 genes. In humans, MDM2 protein is encoded by the MDM2 gene and localized in chromosome 12q13-14 [5]. MDM2 is a nuclear phosphoprotein that binds and inhibits transactivation by tumor protein p53, as part of an autoregulatory negative feedback loop [6]. It binds to p53 via an N-terminal hydrophobic pocket, and this domain contains the highest identity at the amino acid level. The MDM2 p53-binding domains occlude an N-terminal alpha-helix of p53. This prevents the recruitment of transcriptional coactivators and thereby inhibits p53 transactivation function. This transcriptional antagonism can take place within the nucleus, as MDM2 has been detected at p53-responsive promoter elements in chromatin [7]. However, MDM2 is most abundant in the cytosol in many cell lines, suggesting that cytoplasmic localization is important for their function [8, 9]. MDM2 both functions as an E3 ubiquitin ligase that recognizes the N-terminal transactivation domain (TAD) of the p53 and can inhibit p53 transcriptional activation [10]. This protein also affects the cell cycle, apoptosis, and tumorigenesis through interactions with other proteins, including retinoblastoma 1 and ribosomal protein L5. Overexpression of this gene can result in excessive inactivation of tumor protein p53, diminishing its tumor suppressor function. The MDM2 proteins are deregulated in many human cancers and exert their oncogenic activity predominantly by inhibiting the p53 tumor suppressor [11]. Several human tumor types have been shown to have increased levels of MDM2, including soft tissue sarcomas, bladder cancers, and osteosarcomas as well as breast tumors [12–15].

Diagnosis of HCC was considered as a terminal situation and the leading cause of death in cirrhotic patients [16]. However, when diagnosis is achieved at an early stage, effective therapies that improve long term survival are more likely to be achieved [17]. In such sense, the early diagnosis of HCC is crucial for the treatment of patients. In this study, MDM2 was evaluated by immunoassay as a potential TAA in HCC, and autoantibody to this protein was also validated to be an early stage biomarker in immunodiagnosis of HCC.

## 2. Materials and Methods

### 2.1. Sera and General Information

All sera used in this study, including 244 sera from patients with HCC, 30 sera from patients with liver cirrhosis (LC), 30 sera from patients with chronic hepatitis (CH), and 89 normal human sera (NHS), as well as some sera from four HCC patients with serial bleeding samples, were obtained from the serum bank of Cancer Autoimmunity and Epidemiology Research Laboratory at UTEP (University of Texas at El Paso). This study was approved by the Institutional Review Board of UTEP and collaborating institutions.

All HCC patients were diagnosed according to the criteria described in a previous study [18] and had not received treatment with any chemotherapy or radiotherapy. Patients with CH and LC were followed up at least 18 months after collecting blood to exclude individuals with primary biliary cirrhosis and asymptomatic or clinically undetectable HCC. Normal human sera were collected from individuals at the same locality during annual health examinations, who had no obvious evidence of malignancy.

### 2.2. Recombinant Proteins and Antibodies Used in This Study

MDM2 construct pGEX-4T MDM2 WT (plasmid ID: 16237) purchased from Addgene.com was subcloned into pET28a vector producing a fusion protein with NH-terminal 6x histidine and T7 epitope tags. The recombinant protein expressed in* Escherichia coli* BL21 (DE3) was purified using nickel column chromatography. Polyclonal anti-MDM2 rabbit antibody and monoclonal anti-*β*-actin mouse antibody were purchased (Cell Signaling Technology, Inc., Danvers, MA). Horseradish peroxidase- (HRP-) conjugated goat anti-human IgG, HRP-conjugated goat anti-rabbit IgG, HRP-conjugated goat anti-mouse IgG, and FITC-conjugated goat anti-human IgG were purchased (Santa Cruz Biotechnology, Inc., Santa Cruz, CA). Anti-rabbit IgG Fab2 (Alexa Fluor 488) was purchased (Cell Signaling Technology, Inc., Danvers, MA).

### 2.3. Enzyme-Linked Immunosorbent Assay (ELISA)

Standard protocol for ELISA was used as described in our previous study [19, 20]. In brief, a 96-well microtiter plate (ImmunoChemistry Technologies, LLC, Bloomington, MN) was coated overnight at 4°C with recombinant MDM2 protein at a final concentration of 0.5 *μ*g/mL in phosphate-buffered saline (PBS). The antigen-coated wells were blocked with gelatin postcoating solution at room temperature for 2 h. Human sera diluted at 1 : 100 with serum diluent were incubated for 2 h at room temperature in the antigen-coated wells, followed by HRP-conjugated goat anti-human IgG (Caltag Laboratories, San Francisco, CA) at 1 : 4000 dilution. The substrate 2,2′-azino-bis-3-ethylbenzothiazoline-6-sulfonic acid (ABTS, Sigma-Aldrich, St. Louis, MO) was used as detecting reagent. The average optical density (OD) value at a wavelength of 405 nm was used for data analysis. The cutoff value designating positive reaction was the mean optical density (OD) of 89 normal human sera plus 3 standard deviations (SD).

### 2.4. Western Blotting

Denatured recombinant MDM2 protein and cancer cell lysates were electrophoresed on 10% SDS-PAGE and transferred to nitrocellulose papers, respectively. After blocking in PBS with 5% nonfat milk and 0.05% Tween-20 for 1 h at room temperature, the nitrocellulose papers were incubated overnight at 4°C with 1 : 200 dilution of human sera, 1 : 500 dilution of polyclonal anti-MDM2 antibody, and 1 : 500 dilution of monoclonal anti-*β*-actin mouse antibody, separately. HRP-conjugated goat anti-human IgG, HRP-conjugated goat anti-rabbit IgG, and HRP-conjugated goat anti-mouse IgG were, respectively, applied as secondary antibody at a 1 : 10,000 dilution. The ECL-kit was used to detect immunoreactive bands according to the manufacturer's instructions (Thermo Scientific, Waltham, MA).

### 2.5. Indirect Immunofluorescence Assay (IIFA) and Confocal Microscopy

Indirect immunofluorescence assay was performed on Hep2 antinuclear antigen tissue slides (Bion Enterprises, Des Plaines, IL). The sera were diluted at 1 : 80 in PBS and pH 7.4 and incubated with the slides for 30 min at room temperature. After extensive washing, the slides were incubated with fluorescein isothiocyanate- (FITC-) conjugated goat anti-human IgG secondary antibody (Santa Cruz Biotechnology, CA) or anti-rabbit IgG Fab2 (Alexa Fluor 488) as secondary antibody diluted 1 : 100 in PBS for 1 h at room temperature. The slides were washed two times with PBS before adding a drop of mounting media containing 1.5 *μ*g/mL 4′,6′-diamidino-2-phenylindole (DAPI) (Vector Laboratories Inc., Burlingame, CA) to prevent photobleaching. The slides were then examined under fluorescence microscopy, LSM 700 Confocal Microscopy (Zeiss), at 400x magnification. Zen 2009 software was used for images capture and analysis.

### 2.6. Absorption of Antibodies with Recombinant Protein

The diluted human sera (1 : 80) were incubated with recombinant protein (final concentration of recombinant protein in the diluted human sera was 0.03 *μ*g/*μ*L) overnight at 4°C and then centrifuged at 10,000 ×g for 10 min. The supernatant was used for immunofluorescence assay.

### 2.7. Immunohistochemistry (IHC) with Tissue Array Slides

Liver cancer tissue array slide with normal tissue controls (38 cases/80 cores, including pathological diagnosis and pathological grades, two normal liver tissues as controls) was purchased (US Biomax, Inc., Rockville, MD) and used to detect the expression of the MDM2 protein. Tissue array slides were deparaffinized with xylene and dehydrated with ethanol. Antigen retrieval was performed by microwave-heating methods in Trilogy pretreatment solution for 20 min. Avidin/biotin blocking solution was used to prevent nonspecific binding of antibodies. The sections were incubated with polyclonal anti-MDM2 antibody (1 : 50 dilution) for overnight at 4°C. HRP Detection System (HRP streptavidin label and polyvalent biotinylated link) and DAB Substrate Kit were used as detecting reagents. After counterstaining with hematoxylin, the sections were dehydrated and mounted. The slides were observed by light microscopy (Leica DM1000, Germany).

### 2.8. Statistical Analysis

The mean OD value of each group of patients' sera was compared using the Mann-Whitney* U* test; the frequency of autoantibody to TAAs in each group of patients' sera and the expression profile of MDM2 in liver cancer and normal tissue groups were compared using the Chi-square (*χ* ^2^) test with Fisher's exact test, and two significant levels (0.05 and 0.01) were used.

## 3. Results

### 3.1. Frequency and Titer of Autoantibodies against MDM2 in HCC

**Figure 1:**
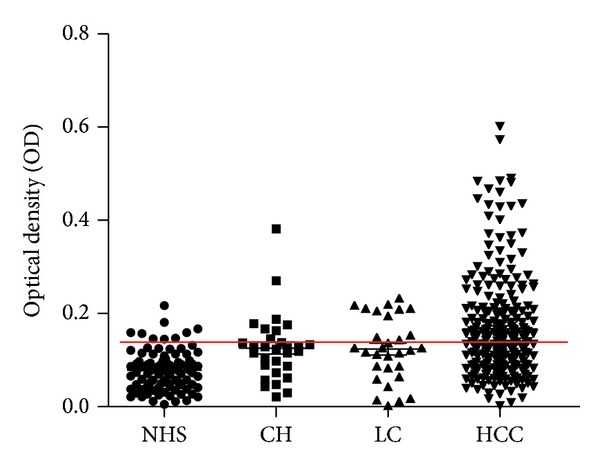
Titer of autoantibody against MDM2 in human sera by ELISA. The range of antibody titers to MDM2 was expressed as optical density (OD) obtained from ELISA. The mean + 3SD of NHS was shown in relationship to all serum samples. Titer of anti- MDM2 in HCC was much higher than that in other types of sera (*P* < 0.01). The cutoff value line for positive samples is indicated with red color.

**Figure 2:**
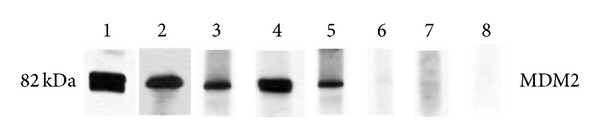
Western blot analysis with representative sera in ELISA. Lane 1, the polyclonal anti-MDM2 antibody was used as positive control. Lanes 2–5, four representative HCC sera which were positive in ELISA also had strong reactivity with MDM2 recombinant protein in Western blot analysis. Lanes 6–8, three randomly selected NHS had negative reactivity with MDM2 recombinant protein.

**Figure 3:**
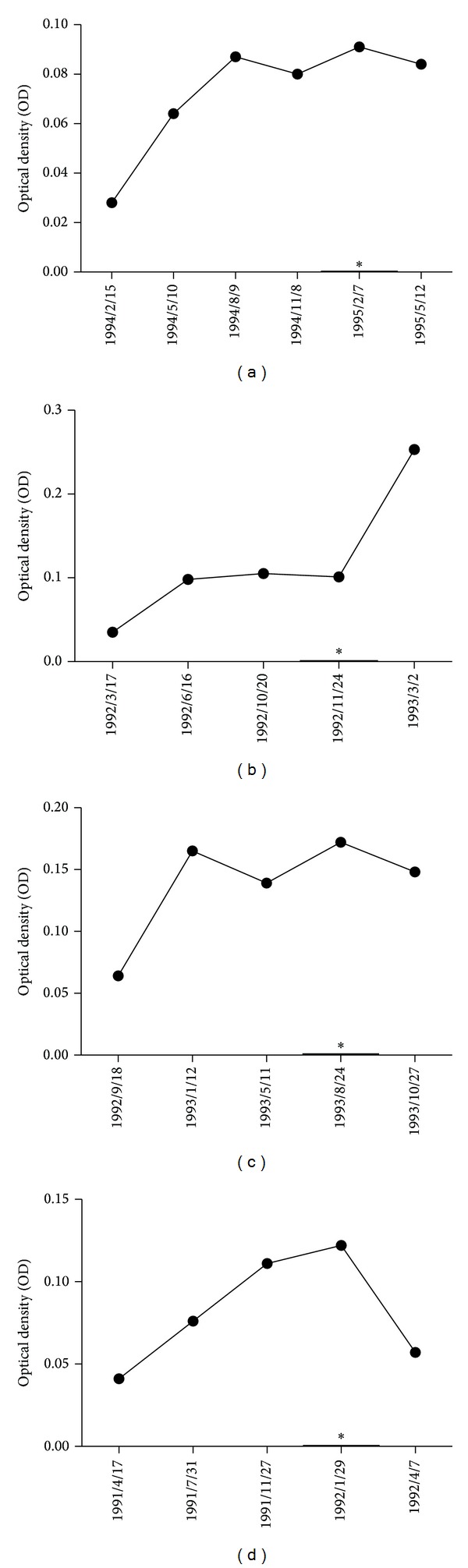
Autoantibody to MDM2 in serial serum samples from four HCC patients (cases 1, 2, 3, and 4). (a) First HCC patient (case 1) was diagnosed on 02/07/1995 with high titer of anti-MDM2 autoantibody. Totally, we had six serum samples within more than one year of time span collected from this HCC patient. The titer of anti-MDM2 autoantibody became higher at month 9 before HCC was detected. (b) Total of five serum samples were acquired from the second HCC patient (case 2). The titer of anti-MDM2 autoantibody became high at month 6 before the date (11/24/1992) of HCC diagnosed. There was low titer of anti-MDM2 autoantibody before 03/17/1992. (c) The third HCC patient (case 3) was diagnosed at the date of 08/24/1993, with a high titer of anti-MDM2 autoantibody. During one-year period (09/18/1992 to 10/27/1993), six serum samples collected from this patient showed that the titer of anti-MDM2 autoantibody became higher at month 6 before HCC was detected. (d) The fourth HCC patient (case 4) was diagnosed at the date of 01/29/1992, and five serum samples were acquired for test. From 04/17/1991 to 01/29/1992 (the date of detection), a gradually increased titer of anti-MDM2 autoantibody can be seen. It became high at month 6 before HCC was detected. The asterisk means the date of HCC diagnosed.

The full-length recombinant MDM2 protein was used as coating antigen in ELISA to screen autoantibodies against MDM2 in sera from patients with HCC, LC, CH, and NHS. In total, 244 sera from patients with HCC, 30 from LC, 30 from CH, and 89 sera from normal human individuals were used in this study. As shown in Table 1, the prevalence of autoantibody against MDM2 was 19.3% (47/244) in HCC, which was significantly higher than that in LC (*P* = 0.000), CH (*P* = 0.002), and NHS (*P* = 0.000). Titer of anti-MDM2 antibodies in human sera is shown in Figure 1. The average titer of autoantibody against MDM2 in HCC sera was higher than that in LC, CH, and NHS (*P* < 0.01). The ELISA results were also confirmed by Western blot analysis.  Figure 2 shows that representative HCC sera with positive reaction to MDM2 in ELISA also have strong reactivity in Western blotting compared to the normal sera. Autoantibody to MDM2 in serial serum samples from four HCC patients (case 1 to case 4) was also tested. The ELISA results were shown in Figure 3. In HCC case 1, anti-MDM2 autoantibody titer was high in nine months before HCC was diagnosed. In HCC case 2, case 3, and case 4, anti-MDM2 autoantibody titer became higher at six months before HCC was detected.

### 3.2. Intense Nuclear Staining Pattern Shown in Hep2 Cells by Indirect Immunofluorescence Assay with Representative Positive HCC Sera

**Figure 4:**
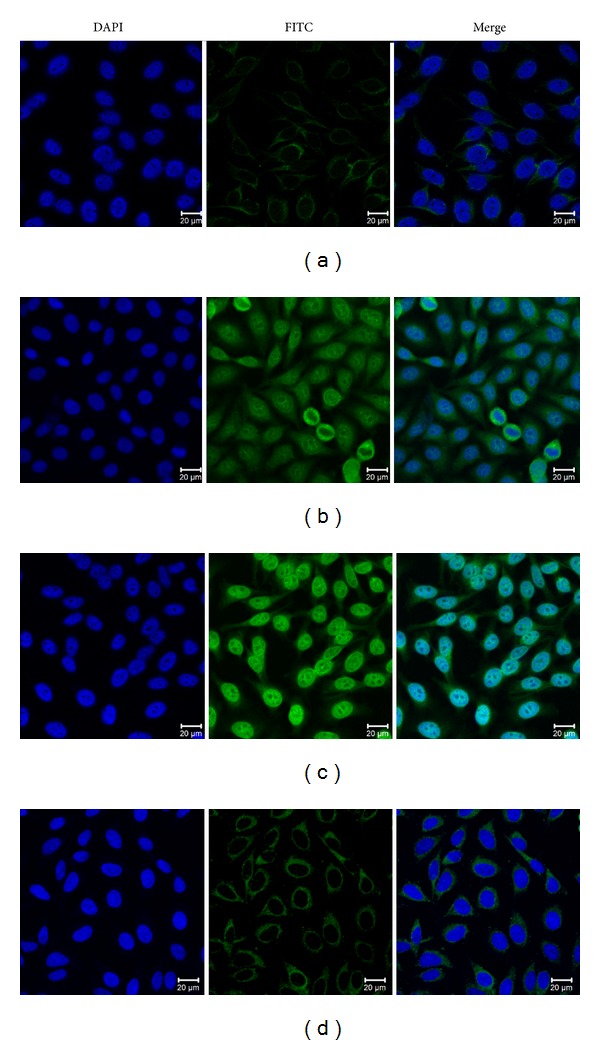
Representative immunofluorescence staining pattern of anti-MDM2 autoantibody positive HCC serum (performed on Hep2 antinuclear antigen tissue slides). (a) NHS were used as negative control; (b) polyclonal anti-MDM2 antibody which showed a nuclear immunofluorescence staining pattern was used as positive control; (c) a representative anti-MDM2 autoantibody positive HCC serum demonstrated an intense nuclear staining pattern; (d) the same HCC serum used in panel (c) was preabsorbed with recombinant MDM2. The nuclear fluorescent staining was significantly reduced.

To further confirm the reactivity of autoantibody in HCC sera to MDM2 and the intracellular location of MDM2, commercially purchased Hep2 cell slides were used in indirect immunofluorescence assay to detect HCC sera with anti-MDM2 positive in ELISA. As shown in Figure 4, a representative anti-MDM2 positive HCC serum had an intense nuclear staining pattern, which was similar in fluorescent staining pattern and cellular location to that shown by polyclonal anti-MDM2 antibody. The fluorescent staining was significantly reduced when the same HCC serum was preabsorbed with recombinant MDM2 protein.

### 3.3. Expression of MDM2 in Liver Cancer Tissues and Normal Hepatic Tissues by Immunohistochemistry

**Figure 5:**
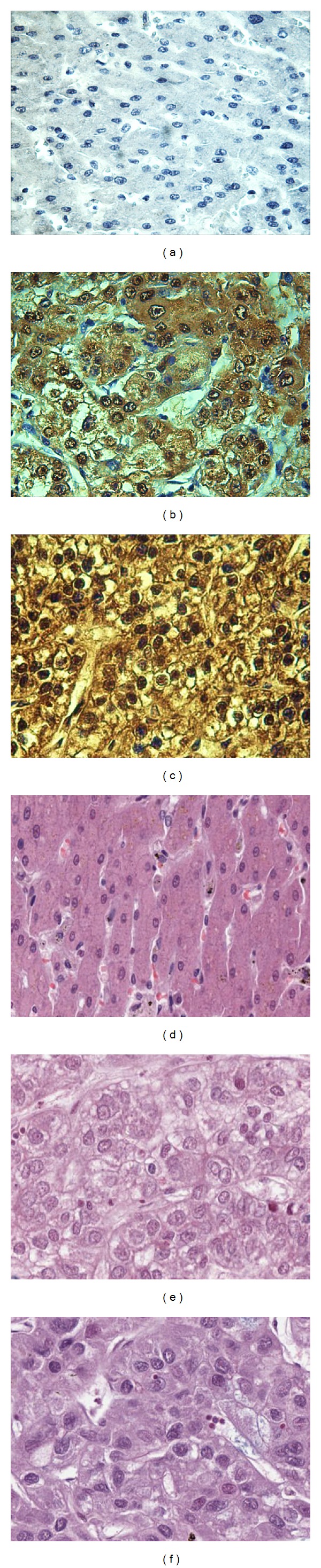
Expression of MDM2 in liver cancer and normal hepatic tissues by immunohistochemistry. The polyclonal anti-MDM2 antibody was used as primary antibody to detect the expression of MDM2 in liver cancer and normal hepatic tissues. (a) A normal hepatic tissue had negative staining; (b) HCC tissue (Grade II) had positive staining; (c) HCC tissue (Grade III) had positive staining; (d) a normal hepatic tissue with HE staining; (e) HCC tissue (Grade II) with HE staining; (f) HCC tissue (Grade III) with HE staining. Grade II: moderately differentiated, cells appear slightly different from normal. Grade III: poorly differentiated, cells appear abnormal and tend to grow and spread more aggressively.

In the current study, the expression profile of MDM2 in liver cancer tissues and normal liver tissues was examined by immunohistochemistry with tissue array slides. Tissue array slides were commercially available for this study, including 30 HCC tissue specimens and 10 normal hepatic tissue specimens. The polyclonal anti-MDM2 antibody was used as primary antibody to detect the expression of MDM2 in liver cancer and normal hepatic tissues. As a result, 24 of the 30 HCC tissues were positively stained (80.0%). None of the 10 normal hepatic tissues were positively stained (0%). The characteristics of patients and MDM2 expression in liver cancer are shown in Table 2. The expression of MDM2 in liver cancer and normal hepatic tissues is shown in Figure 5.

## 4. Discussion

Many studies demonstrated that sera from patients with cancer contain antibodies, which react with a unique group of autologous cellular antigens generally known as tumor-associated antigens (TAAs). During the progression from chronic liver disease to HCC, the novel autoantibodies appearing with malignant transformation will be more likely to be related to events associated with tumorigenesis, and therefore these autoantibodies can be used as reporters identifying aberrant cellular mechanisms in tumorigenesis and also serve as immunodiagnostic markers for HCC detection [21–23]. As we know, AFP is the most common and traditional biomarker being used for diagnosis of HCC in current clinical screening. However, both its sensitivity and specificity are limited. There are still about 40% of HCC patients that cannot be identified by this approach. HCC patients with small tumors or with well-to-moderately differentiated tumors may not have high level of serum AFP. The ultrasonic and computer tomography are useful when the tumors are big enough to be detected. Usually it is too late for patients to get effective therapy when the HCC is detected by these methods. Therefore, identification of a better biomarker for the early stage HCC is crucial for reducing the HCC mortality rate in the population. Anti-TAAs autoantibodies, which have been called “reporters” from the immune system, can be used as biomarkers in immunodiagnosis of early stage HCC [24]. In our previous study, over ten oncoproteins have been evaluated and validated as TAAs in HCC, and autoantibodies against these TAAs have been detected in sera from patients with HCC [25]. But the sensitivity and specificity of autoantibodies to single TAA as a diagnostic marker in HCC are not high enough for the diagnosis of HCC [26]. The method to solve this problem is to combine the known TAAs making a TAA array with multiple TAAs, by which a higher sensitivity and specificity can be acquired, but it is still not good enough till now. It is necessary to work on the validation of TAAs which are good at early stage diagnosis in HCC patients.

The MDM2 protein plays a critical role in the negative regulation of p53. MDM2 can lead to nuclear export, ubiquitination, and nuclear and cytoplasmic proteasomal degradation of p53 through directly binding to the p53 transactivation domain [27, 28]. p53-mediated tumor suppressing activity can be inhibited by high levels of MDM2 through p53 inactivation [29]. The MDM2 protein also interacts with several cell cycle regulatory proteins that may contribute to its tumorigenic ability. So the expression level of MDM2 has close relationship with tumorigenesis [30]. The role of MDM2 in HCC and its overexpression in HCC patients are studied by many researchers. A study on Japanese patients with HCV infection showed that MDM2 gene was significantly associated with development and recurrence of HCC [31]. MDM2 SNP309 plays a major role in the carcinogenesis of HCC, especially among Caucasian populations [29]. The findings from different areas support that MDM2 is significantly associated with increased risk of hepatocellular carcinoma [32–35]. MDM2 SNP309 G allele is a susceptibility gene for the development of viral hepatitis-related hepatocellular carcinoma [36–38]. The combination of MDM2 SNP 309 and TP53 Arg72Pro genotypes confers higher risk to develop HCC [39–41]. The MDM2 gene silenced by shRNA effectively inhibits HCC tumorigenesis of subcutaneously xenografted HepG2 cells in nude mice [42]. MDM2 antagonist can inhibit tumor growth in HCC with different types of p53 in vitro [43]. HCC patients with a low expression of MDM2 survived significantly longer as compared with patients with high expression [44]. The expressions of MDM2 protein and gene were also related to the high invasiveness of HCC through inactivating the tumor-suppressor function of the p53 gene [45, 46]. MDM2 was considered to be a valuable target for cancer therapy and MDM2 blockade with suitable antagonists was shown to block tumor growth in a number of models [47]. MDM2 overexpression was also considered to be a useful predictor of poor prognosis in patients with HCC following hepatic resection [48]. However, there is nothing reported about the possibility of MDM2 as a biomarker of cancer. MDM2 is predominantly distributed in the cell nucleus, and some of MDM2 can be detected in the cytoplasm. The expression of MDM2 can be increased during the tumorigenesis. On the situation of cell injury or the high penetration of the cell membrane, the immune system can find the exposed MDM2 protein and the autoantibodies will appear in the serum. Since MDM2 is overexpressed in HCC, we try to evaluate whether the MDM2 protein is a potential TAA in HCC and validate if autoantibody to this protein can be used as an early stage biomarker in immunodiagnosis of HCC.

In our present study, the results indicated that almost twenty percent of HCC sera showed immune response to MDM2 recombinant protein. The mean titer of autoantibodies against MDM2 in sera from patients with HCC was significantly higher than that in LC, CH, and normal individuals. The results were also confirmed by Western blot analysis, which showed that representative HCC sera with positive reaction to MDM2 in ELISA also have strong reactivity compared to normal sera. In the further study, anti-MDM2 autoantibodies were detected in sera from several HCC patients with serial bleeding samples. A high titer of autoantibodies against MDM2 in ELISA can be seen in sera in 6 months to 9 months before the clinical diagnosis of HCC. Most cases of HCC are secondary to either a chronic viral hepatitis or cirrhosis. HCC develops when there is a gene mutation to the cellular machinery which causes the cell to replicate at a high rate, which results in the cell avoiding of apoptosis. Constant cycle of damage followed by repair can lead to mistakes during DNA repair which in turn lead to carcinogenesis. During the course of HCC formation, some new presenting autoantibodies maybe have the value for diagnosis of HCC patients with an early stage of tumorigenesis. Anti-MDM2 autoantibodies are potential biomarkers for immunodiagnosis of HCC patients due to the low positive rate in LC, CH, and normal individuals and higher positive rate in HCC patients.

It has been reported that MDM2 protein is overexpressed in HCC tissue. In a previous report by MF Zhang's group, MDM2 expression was examined in 181 pairs of HCC tissues and the adjacent hepatic tissues by using immunohistochemistry, through which the group found that MDM2 was overexpressed in all the HCC cases [49]. But in another study, the result of immunohistochemistry showed that MDM2 was expressed in 26% of HCC, and its expression correlated positively with p53 mutations [50]. In our present study, eighty percent of HCC liver tissues were positively stained with anti-MDM2 antibody and there were no differences between HCC tissues with Grade III and Grade II in using immunohistochemistry approach with HCC tissue array slide. IHC study with HCC tissue array has verified that the cellular localization of MDM2 is found in the nucleus of cell. We also verified the cellular localization of MDM2 by indirect immunofluorescence assay, and anti-MDM2 autoantibody positive HCC serum demonstrated an intense nuclear staining pattern. The cellular localization of MDM2 is associated with its oncoprotein function as negative regulator of tumor suppressor protein p53.

MDM2 plays an important role in HCC tumorigenesis. Data from a Chinese population shows that the MDM2 indel polymorphism may be a genetic modifier for developing HCC [51]. So MDM2 protein maybe appeared in early stage of HCC generation and its antibodies have the possibility of early biomarker in HCC patients. Our current study focused on the possibility of its role as a biomarker in early stage HCC diagnosis. The data from our study has demonstrated that MDM2 is a potential TAA in HCC, and the anti-MDM2 autoantibodies may be used as a biomarker in early stage HCC diagnosis because a higher titer of autoantibodies against MDM2 in ELISA can be seen in sera at 6 months to 9 months before the clinical diagnosis of HCC. The limitation of our study is that no information of these HCC patients with serial bleeding samples was acquired after the HCC diagnosis. We did not know the reason why the titer of autoantibodies against MDM2 decreased after diagnosis. The affecting factors may range from the surgery procedures, the drug used by the patients, the chemotherapy, or the radiotherapy. Therefore, in our future study more cases with complete clinical data should be included in order to further investigate the correlation of the role of hepatic tissue MDM2 protein expression and the presence of elevated MDM2 autoantibodies in serum of patients with HCC, to be evaluated as early biomarkers in immunodiagnostic of HCC.

## Figures and Tables

**Table 1:** Frequency of autoantibody against MDM2 in human sera by ELISA.

Type of sera	Number tested	Autoantibody to MDM2 (%)
HCC	244	47 (19.3)
LC	30	0**
CH	30	2 (6.6)**
NHS	89	0**

The cutoff value designating positive reaction was the mean optical density (OD) of 89 normal human sera plus 3 standard deviations (SD); *P* value relative to NHS (*P* = 0.000), CH (*P* = 0.002), and LC (*P* = 0.000), **P < 0.01; HCC: hepatocellular carcinoma; LC: liver cirrhosis; CH: chronic hepatitis; and NHS: normal human sera.

**Table 2:** Characteristics of patients and MDM2 expression in liver cancer.

Variable		Frequency	%
Age	≥60	7	23.3
	<60	23	76.7
Gender	Male	20	66.7
	Female	10	33.3
Grade	II	15	50.0
	III	15	50.0
Normal liver tissue	Negative	10	100.0
	Positive	0	0.0
Liver cancer	Negative	6	20.0
	Positive	24	80.0**

**P value of liver cancer to normal tissue: *P* < 0.01.
